# Effects of stand structural diversity on carbon storage of Masson pine forests in Fengyang Mountain Nature Reserve, China

**DOI:** 10.48130/forres-0025-0010

**Published:** 2025-06-06

**Authors:** Yongzhao Miao, Ran Tong, Nianfu Zhu, Song Chen, Fang Zhou, G. Geoff Wang, Tonggui Wu

**Affiliations:** 1 East China Coastal Forest Ecosystem Long-term Research Station, Research Institute of Subtropical Forestry, Chinese Academy of Forestry, Hangzhou 311400, China; 2 College of Landscape Architecture, Nanjing Forestry University, Nanjing 210037, China; 3 Department of Forestry and Environment Conservation, Clemson University, Clemson, SC 29634-0317, USA

**Keywords:** Spatial structural diversity, Non-spatial structural diversity, Carbon storage, Carbon pool components, Masson pine forests

## Abstract

Stand structural diversity, encompassing spatial and non-spatial dimensions, is a key indicator of forest carbon storage, yet its relative impacts on multiple carbon pools remain unclear. Additionally, whether structural diversity consistently influences carbon storage across overstory, understory, and soil layers is uncertain. This study examined carbon storage dynamics across 13 secondary Masson pine forests within the Fengyang Mountain Nature Reserve. Principal component analysis was used to classify the stands into three types based on their spatial and non-spatial structural diversity: Type I (high spatial and high non-spatial diversity), Type II (high spatial but low non-spatial diversity), and Type III (low spatial and low non-spatial diversity). Total carbon storage was highest in Type I, while carbon storage in the understory layers was lowest in this type. Spatial structural diversity had a stronger influence on carbon storage than non-spatial diversity, with the uniform angle index primarily affecting overstory carbon storage, and the crowding index influenced understory carbon storage. Random forest analysis identified biomass and structural diversity as major predictors of carbon storage. Partial least squares path modeling revealed that spatial structural diversity indirectly increased overstory and soil carbon storage, but reduced understory carbon storage by modulating biomass. Our results highlight that spatial structural diversity is a dominant driver of carbon storage in forest ecosystems, with contrasting effects on overstory, understory, and soil layers, underscoring its critical role in regulating forest carbon dynamics.

## Introduction

The configuration of forest stand structure significantly influences the stability, functional diversity, and productivity of stands, which in turn shapes the diversity and function of ecosystems^[1−4]^. In recent years, numerous studies have highlighted the key role of stand structural diversity in forest functions, such as biomass and carbon stocks, noting that its impact may exceed even species diversity^[5−7]^. Forest stand structure is a complex and multi-dimensional concept, being divided into spatial structure and non-spatial structure^[8−10]^. Non-spatial structure encompasses aspects such as diameter structure, tree height structure, stand density, etc., with diameter structure being particularly significant in influencing resource allocation and competition within forests. Additionally, spatial structure is focused on the spatial distribution pattern of individual trees and the distribution of their associated attributes, such as tree species composition and spatial isolation, etc^[11]^. These factors further regulate forest productivity and carbon fixation ability by affecting the acquisition of light, water, and nutrients^[12,13]^.

Previous studies have shown that stand structural diversity can directly increase above-ground biomass or enhance it through complementary effects, which has been interpreted as a result of niche differentiation, and promotion^[14,15]^. For instance, complex forest stand structure enables organisms to occupy more spatial niches and larger canopy packages. Moreover, refining the forest stand structure of the overstory not only improves light and nutrient utilization, but also regulates carbon storage throughout the entire forest ecosystem by influencing understory vegetation, litter dynamics, and soil processes^[16−18]^. It remains an open question in current research whether spatial or non-spatial structure plays a more dominant role in shaping carbon storage.

Forest ecosystems play a crucial role in the global carbon cycle, sequestering approximately half of the carbon stored in terrestrial ecosystems^[19]^. Although the majority of carbon is stored in the tree and soil layers, the shrub, herb, and litter layers also contribute notably to ecosystem carbon dynamics^[20,21]^. The tree layer, with its vertical dominance, efficiently captures light and soil resources, resulting in greater carbon accumulation. In contrast, the understory layer receives less light and nutrients, limiting its carbon input. Stand structural diversity, shaped by species composition, spatial arrangement, soil properties, and microbial activity, influence how carbon is distributed and stored across these different layers. However, most existing studies have concentrated on the tree layer, overlooking how spatial structure affects the carbon storage of other components. Furthermore, previous findings remain inconclusive, with some studies suggesting that increased spatial heterogeneity enhances carbon sequestration through niche complementarity, while others indicate diminishing returns due to intensified competition^[22−24]^. These inconsistencies and knowledge gaps underscore the need for a more integrative approach. Therefore, our study aims to assess whether stand structural diversity promotes consistent carbon storage across all forest components, or leads to divergent patterns, offering a clearer understanding of the structural mechanisms regulating ecosystem carbon stocks.

Masson pine (*Pinus massoniana* Lamb.) is a species widely distributed across China, with its fast growth and strong adaptability, serves as an important resource for carbon sequestration. The forest area of Masson pine exceeds 1.13 million hectares, and its standing stock accounts for 4% of the national total^[25]^. This study concentrates on the impact of forest stand structure on carbon storage for total and individual carbon pools of the secondary forests dominated by Masson pine in the Fengyang Mountain Nature Reserve, located in eastern China. The objectives of the study are to: (a) identify which exerts a stronger influence on carbon storage—stand spatial or non-spatial structural diversity; (b) explore the consistency of the relationship between stand structural diversity and carbon storage for total and individual carbon pools in the forest ecosystem; and (c) analyze the various factors that directly and indirectly affect carbon storage for total and individual carbon pools, and to provide corresponding management suggestions.

## Materials and methods

### Site description and experimental design

The study was conducted in Fengyang Mountain Nature Reserve (27.97° N, 119.14° E), Zhejiang Province, southeast China. The area has a subtropical monsoon climate with a mean temperature of 12.3 °C, and a mean annual precipitation of 2,400 mm. The dominant soil types are red and yellow soils. The study area features Masson pine forests established through aerial seeding on post-fire sites in the 1970s, with relatively consistent initial site conditions across the stands due to landscape homogenization following historical wildfires. Over decades of minimal human disturbance, natural regeneration and succession have fostered the development of a mixed forest, with Masson pine as the dominant species. Associated tree species comprise *Schima superba*, *Pinus taiwanensis*, and *Eurya japonica*. The shrub layer is predominantly composed of *Camellia cuspidata*, *Ilex elmerrilliana*, *I. pubescens*, and *Loropetalum chinense,* etc. The herb layer features *Woodwardia japonica*, *Curculigo orchioides*, *Liriope spicata*, and *Dicranopteris pedata* as the main species.

In August 2023, 13 20 m × 20 m plots were established at the study site, with the basic information for each plot recorded (Supplementary Table S1). A total of 35 different woody plant species were recorded across these plots. The average density of woody plants was approximately 1,200 per hectare, with a range from 700 to 1,650 per hectare. The average diameter at breast height (DBH) was 16.6 cm, ranging from 5.0 to 62.0 cm. The plots were situated at an altitude of about 355 m with an average slope of 33 degrees. Tree samples with ≥ 5 cm diameter at breast height (DBH) were selected to calculate the stand basal area, as well as to analyze both non-spatial structural parameters and spatial structure parameters.

### Stand structural diversity investigation

The intricacy of forest stand structure was evaluated by examining the spatial and non-spatial structure of the woody plants in the sample plots, in conjunction with the corresponding stand structural diversity parameters. The non-spatial structural diversity parameters were derived from DBH measurements—a widely used, cost-effective, and ecologically reliable proxy for tree size. Specifically, we obtained the non-spatial structural diversity of the forest stand with tree DBH ≥ 5 cm using the following metrics: the coefficient of variation (*CV*) index for diameter size variation, skewness (*SK*) index for diameter distribution asymmetry, Gini coefficient (*GC*) index for basal area inequality, and Shannon-wiener (*H*) index for size diversity. The forest spatial structural diversity parameters are based on the relationship of neighboring trees, and describe the average state of the overall forest stand structure. It was characterized using four complementary parameters including uniform angle (*W*), dominance (*U*), mingling (*M*), and crowding (*C*) indexes, each reflecting a distinct spatial dimension such as distribution pattern, size hierarchy, species segregation, and density effect. These indices were selected for their ability to capture unique aspects of spatial organization with minimal functional redundancy^[11]^. Specifically, *W* describes the uniformity of tree distribution; *U* reflects the degree of differentiation in tree diameter at breast height; *M* expresses the segregation level among tree species; and *C* indicates the degree of tree crowding (Supplementary Table S2).

### Sample collection and chemical analysis

In each plot, soil samples were gathered from the 0−20 cm, and 20−40 cm depth layers using the 'S' sampling method with a 5.0 cm diameter soil drill. Approximately 500 g of soil from each layer was evenly collected and then mixed to create a single sample. The soil was stored in zip-lock bags and brought to the laboratory promptly. In the laboratory, the soil samples were divided into two parts: one part was air-dried and passed through 2 mm, and 0.149 mm sieves for chemical property analysis. The other part was stored in a refrigerator at 4 °C for soil microbial biomass, and enzyme activity determination.

Soil bulk density (BD) was determined by the cutting ring method (100 cm^3^), while pH was measured through potentiometry with a soil-to-water ratio of 2.5 to 1. Soil organic carbon (SOC) was determined by the potassium dichromate external heating method. Soil total nitrogen (TN) was measured using the Kjeldahl method. Soil total phosphorus (TP) was determined by the alkaline fusion molybdenum-antimony anti-spectrophotometric method. Soil ammonium nitrogen (NH_4_^+^) was measured by the indophenol blue colorimetric method. Soil nitrate nitrogen (NO_3_^−^) was determined by the cadmium reduction-diazotization coupling colorimetric method. Soil available phosphorus (AP) was measured by the ammonium fluoride-hydrochloric acid extraction molybdenum-antimony anti-spectrophotometric method. The soil enzyme activities, including *β*-glucosidase (*β*G), N-acetyl-*β*-D-glucosaminidase (NAG), leucine aminopeptidase (LAP), acid phosphatase (ACP), and alkaline phosphatase (ALP), were determined using an improved microplate fluorescence method. Microbial biomass indicators were determined using the chloroform fumigation extraction method, including microbial biomass carbon (MBC), microbial biomass nitrogen (MBN), and microbial biomass phosphorus (MBP).

### Carbon storage estimation

#### Vegetation carbon

In each plot, all trees with DBH ≥ 5 cm were measured. Biomass estimation was conducted using compatible biomass models for different tree species or stand types in subtropical areas^[26]^. The total biomass of the arbor layer was obtained by calculating the DBH of the tree species using existing biomass regression models (Supplementary Table S3).

In each plot, three 2 m × 2 m subplots were established for the determination of shrub layer biomass. Within these 2 m × 2 m subplots, 1 m × 1 m subplots were set up to determine the biomass of the herb layer. The above ground and underground parts of the shrub and herb layers was quantified using the total harvest method (collecting all samples above and below ground). The fresh weight of the shrub layer was measured by separating the branches, leaves, and roots, whereas the fresh weight of the herb layer was recorded by distinguishing between the above ground and underground parts. After thorough mixing of the samples within the same plot, a certain amount of each sample was selected for the laboratory analysis. All samples were weighed while fresh, then inactivated enzymatically at 105 °C for 30 min, and dried at 80 °C to a constant weight. Carbon content data was obtained directly from the referenced publication^[27]^. Detailed values for various tree species are provided in Supplementary Table S4.

#### Litter carbon

In each plot, three 1 m × 1 m subplots were set up to determine biomass of the litter layer. All litter within these quadrats was collected using the complete harvest method, and its mass weighed. The litter in each plot was collected, sent to the laboratory, and dried to a constant mass to calculate the moisture content. The carbon stored in the litter layer was calculated by multiplying the calculated litter biomass by the carbon ratio reported by the Chinese forest vegetation (Supplementary Table S4).

#### Soil carbon

Soil carbon stocks are calculated as follows:

$ {C}_{s}=\sum _{i=1}^{\mathrm{n}}\left({S OC}_{i}\times {\mathrm{B}\mathrm{D}}_{i}\times {\mathrm{D}}_{i}\times 10\right) $

where *C*_*S*_ is the soil carbon stock (g·m^−2^), *i* represents the different soil layers, BD*_i_* is the bulk density (g·cm^− 3^) of soil in layer *i*, *SOC*_*i*_ is the organic carbon concentration (g·kg^−1^) of soil in layer *i*, and D*_i_* is the thickness (cm) of the soil layer *i*.

### Statistical analyses

We applied logarithmic transformation to all variables to ensure data normality. The 'FactoMineR' and 'factoextra' packages were utilized for Principal Component Analysis (PCA), to conduct clustering analysis on the sample plot data, following the approach outlined in previous studies^[28,29]^. Given that the Pearson correlation coefficient between *CV* and *GC* indexes is 0.90, *GC* index was chosen as the variable for PCA analysis to minimize data redundancy and enhance analysis efficiency. One-way analysis of variance (ANOVA) was used to compare the differences in soil chemical and microbial properties, stand structural diversity, and carbon pool components across the three structural clusters (Supplementary Table S5). The Tukey's Honest Significant Difference test, implemented using the 'stats' package, was conducted for multiple comparisons to examine the impact of structural clustering on these variables. The Mantel test, facilitated by the 'linkET' package, was used to analyze the interactions between soil chemical and microbial properties, stand structural diversity, and carbon pool components. Random forest (RF) analysis, conducted with the 'randomForest' and 'rfPermute' packages, was used to identify the main predictors of carbon storage among stand structural diversity, soil chemical and microbial properties, and biomass. The model was constructed with 1,000 trees, and variable importance was assessed based on the percent increase in mean squared error (%IncMSE). The significance of each predictor was evaluated through 500 permutation tests, and parallel computation was enabled using four cores. Partial least squares path modeling (PLS-PM), carried out using the 'plspm' package, tested the influences of chemical and microbial properties, stand structural diversity, and biomass on soil carbon pool components. The model structure was defined based on theoretical expectations, and the estimation followed a reflective measurement mode. Model evaluation included the coefficient of determination (R^2^) for each endogenous construct, the significance of path coefficients assessed via bootstrapping (500 resamples), and the overall goodness-of-fit (GoF). Visualization of the inner and outer models was conducted to aid interpretation of the latent structure and indicator loadings. Calculations of stand structural diversity parameters were performed using the 'dplyr' and 'e1071' packages. All analyses were performed in R (version 4.3.1).

## Results

### Clustering based on stand spatial and non-spatial structural diversity

**Figure 1:**
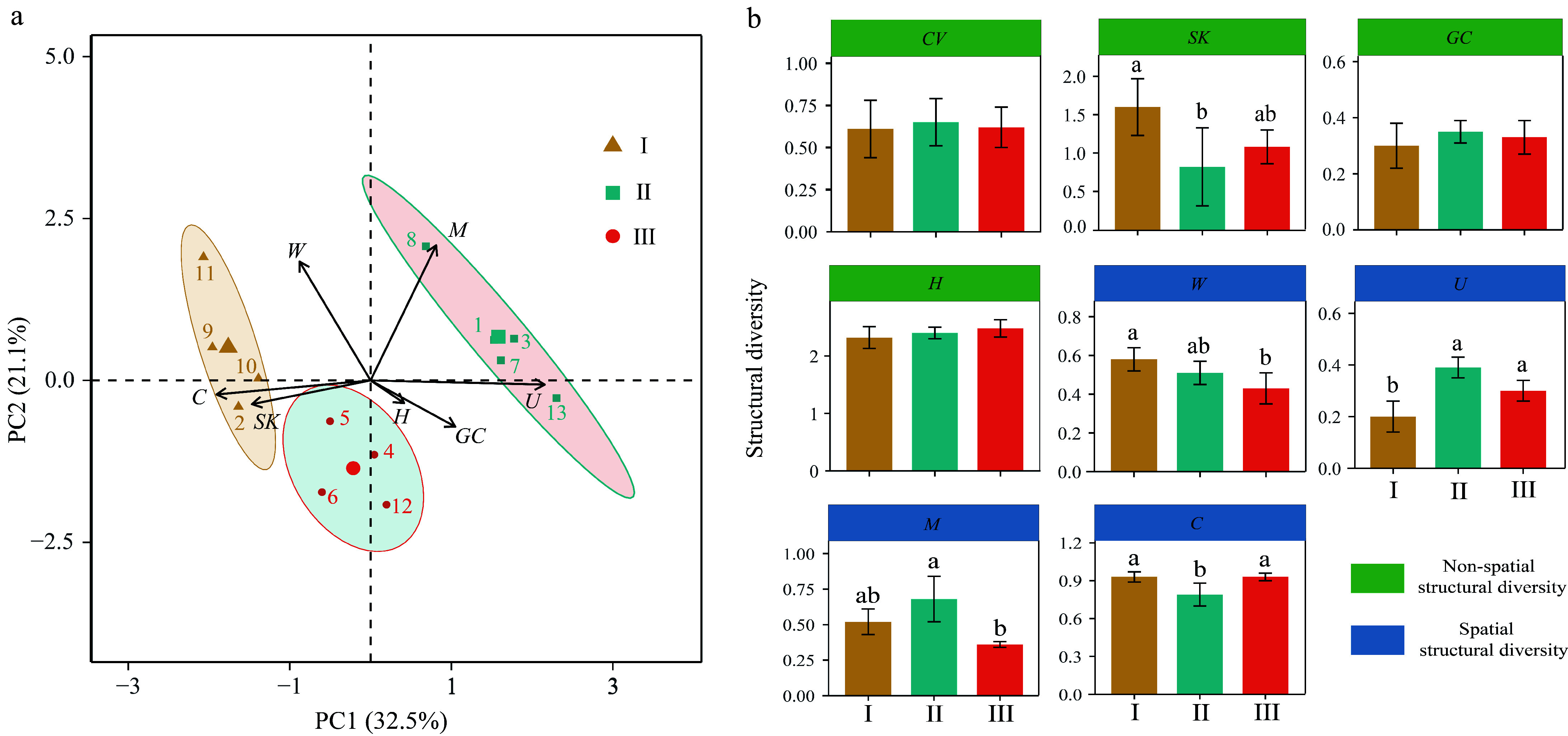
PCA analysis results for (a) forest type classification, and (b) differences in spatial and non-spatial structural diversity among forest types.

The 13 plots were categorized into three stand types based on stand structural diversity, as determined by Principal component analysis (PCA). These categories are as follows: Type I (n = 4) with high spatial and high non-spatial structural diversity, Type II (n = 5) with high spatial and low non-spatial structural diversity, and Type III (n = 4) with low spatial and low non-spatial structural diversity. In addition, the loadings of *C* and *U* indexes were found to be close to the axis of the first principal component (PC1), indicating their substantial contributions to the variance explained by PC1, albeit in opposite directions (Fig. 1a). The *SK* and *C* indexes of Type I were significantly higher than those of Type II. The *W* index of Type I was significantly higher than that of Type II (Fig. 1b).

### Carbon storage across stand types

**Figure 2:**
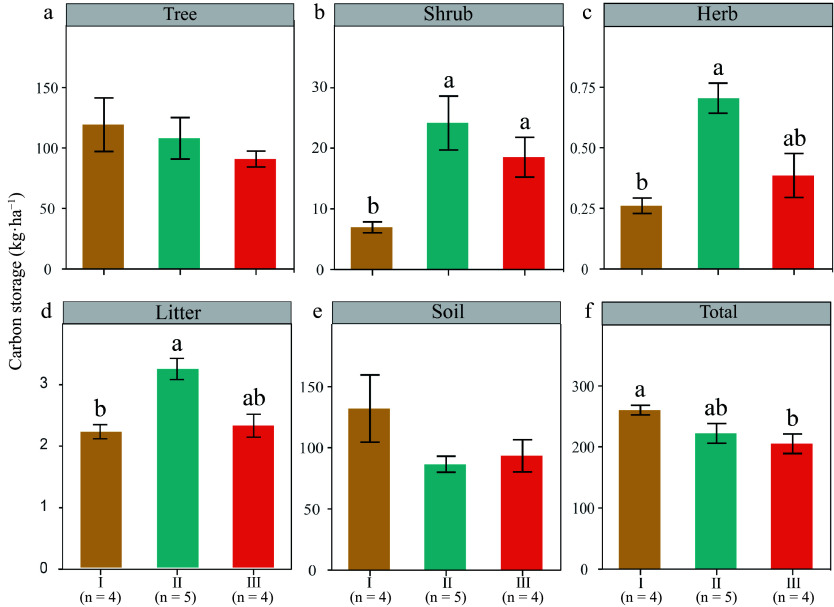
Carbon storages of total and individual carbon pool components across various forest types. Values are presented as mean ± SE (standard error). Lower-case letters denote significant differences at *p* < 0.05. The number of plots for each type is indicated on the figure.

**Figure 3:**
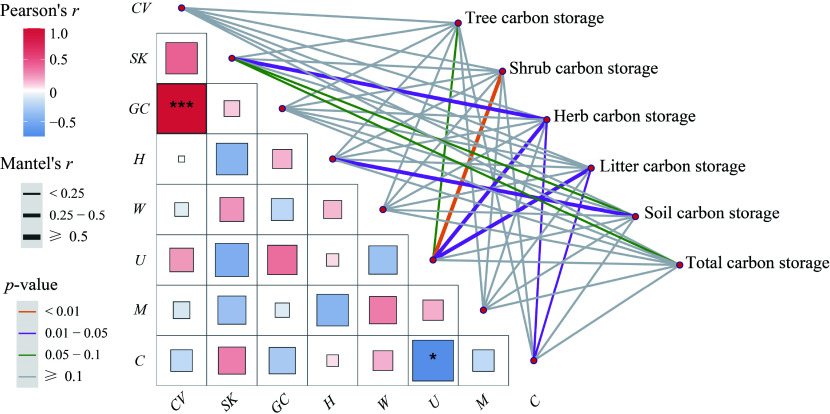
Mantel test between structural diversity and ecosystem carbon pool components and the correlation between structural diversity pairwise. The lines denote significant relationships, while the line width represents Mantel's *r* statistic. Pair-wise correlations between influencing factors are shown in a color gradient matrix. The color represents Pearson's correlation coefficient, which is shown with an asterisk when the result is significant (*p* < 0.05), and *, *** indicate the signiﬁcance level at *p* < 0.05, *p* < 0.001, respectively. The abbreviations of the influencing factors are explained in the Methods section.

The total carbon storage of Type I was significantly greater than that of type III (*p* < 0.05) (Fig. 2a). No significant differences were observed in carbon storage between the tree and soil layers; however, the Type I exhibited the highest levels (Fig. 2b, f). Conversely, the carbon storage in the shrub, herb, and litter layers layer of Type I was significantly lower compared to Type II and Type III (*p* < 0.05, Fig. 3c). The percent of carbon storage in overstory, understory, and soil layers can be seen in Supplementary Fig. S1.

The soil TN content in Type I was significantly higher than that in Type II in the 0−20 cm layer (*p* < 0.05). The soil TP and ACP, and MBC contents in Type I were significantly greater than those in Type III in the 0−20 cm layer (*p* < 0.05). The soil MBN content in Type I was also significantly higher than that of Type II and Type III in the 0−20 cm layer (*p* < 0.05). The soil NH_4_^+^ content in Type I was significantly higher than that in Type II in the 20−40 cm layer (*p* < 0.05) (Supplemental Table S2).

### Relationships between stand structural diversity and carbon storage

The Mantel test elucidated the relationships between carbon storage, stand spatial, and non-spatial structural diversity (Fig. 3). *SK* and *U* indexes were the primary factors influencing carbon storage. In particular, total carbon storage was influenced by the *SK* index, while carbon storage in the tree, shrub, herb, and litter layers was influenced by the *U* index. Additionally, carbon storage in the herb and litter layers was also affected by crowding. The *CV* index was positively correlated with the *GC* index (*p* < 0.001), and the *U* index was negatively correlated with the crowding index (*p* < 0.05).

### Effects of forest stand structure diversity on carbon storage

**Figure 4:**
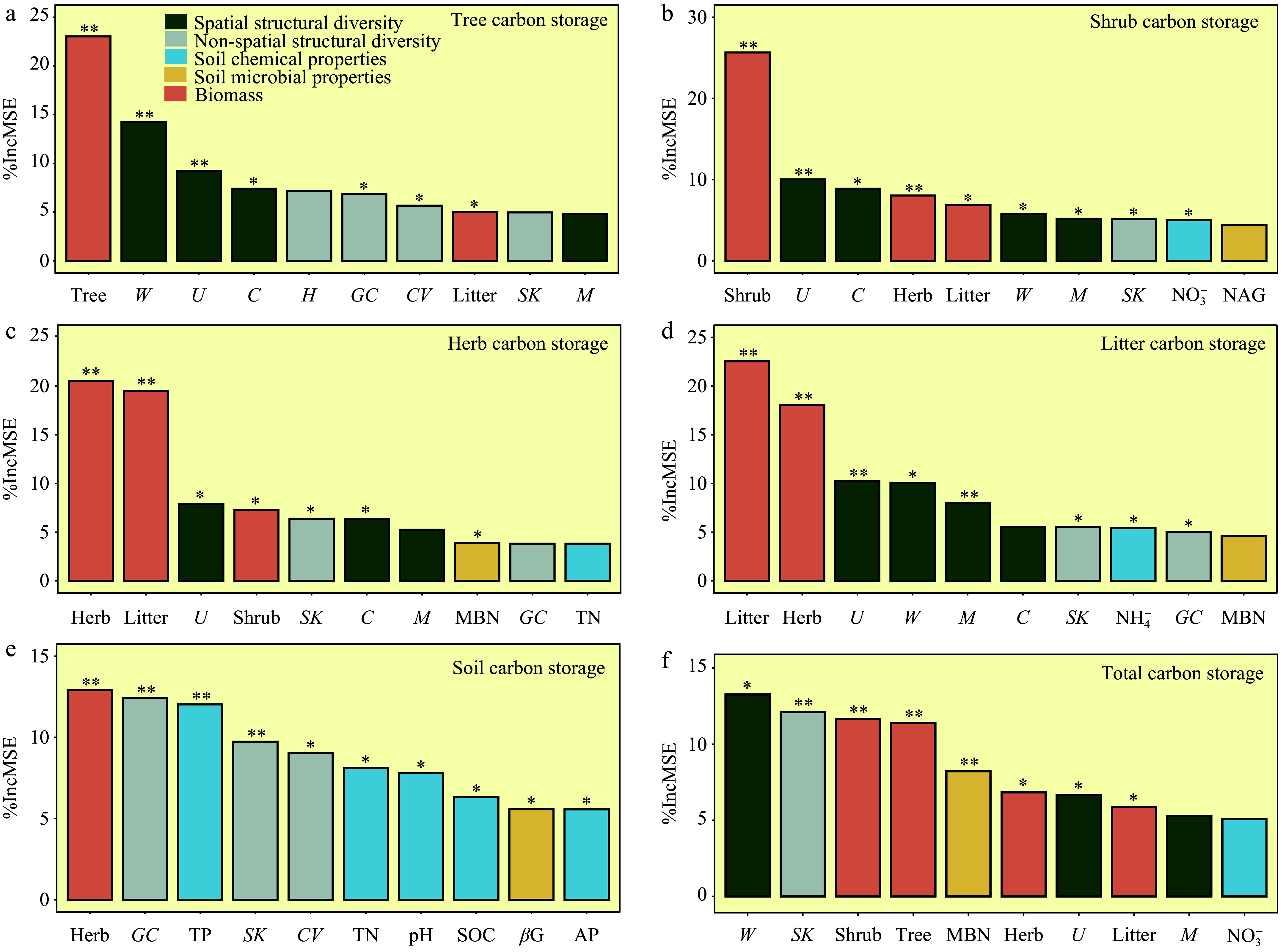
Random Forest analysis identifying key predictors of carbon storage. The results focus on the top ten predictors that critically influence carbon storage in various components: (a) tree, (b) shrub, (c) herb, (d) litter, (e) soil, and (f) total carbon storage. Each panel quantifies the increase in mean squared error (% Increase in MSE) upon the exclusion of these key predictors, where higher values indicate a greater impact on model accuracy. Statistical significance is marked by asterisks, with * for *p* < 0.05, and ** for *p* < 0.01, underscoring the most influential predictors. The abbreviations of the influencing factors are explained in the Methods section.

The random forest analysis further revealed that total carbon storage was most influenced by biomass and stand structural diversity (Fig. 4a). Tree carbon storage was mostly affected by stand spatial and non-spatial structural diversity, with tree biomass showing the highest contributions (Fig. 4b). Carbon storage in shrub, herb, and litter layers is primarily determined by biomass, and is also influenced by stand spatial structural diversity (Fig. 4c−e). Soil carbon storage is primarily influenced by stand non-spatial structural diversity and soil chemical properties (Fig. 4f).

**Figure 5:**
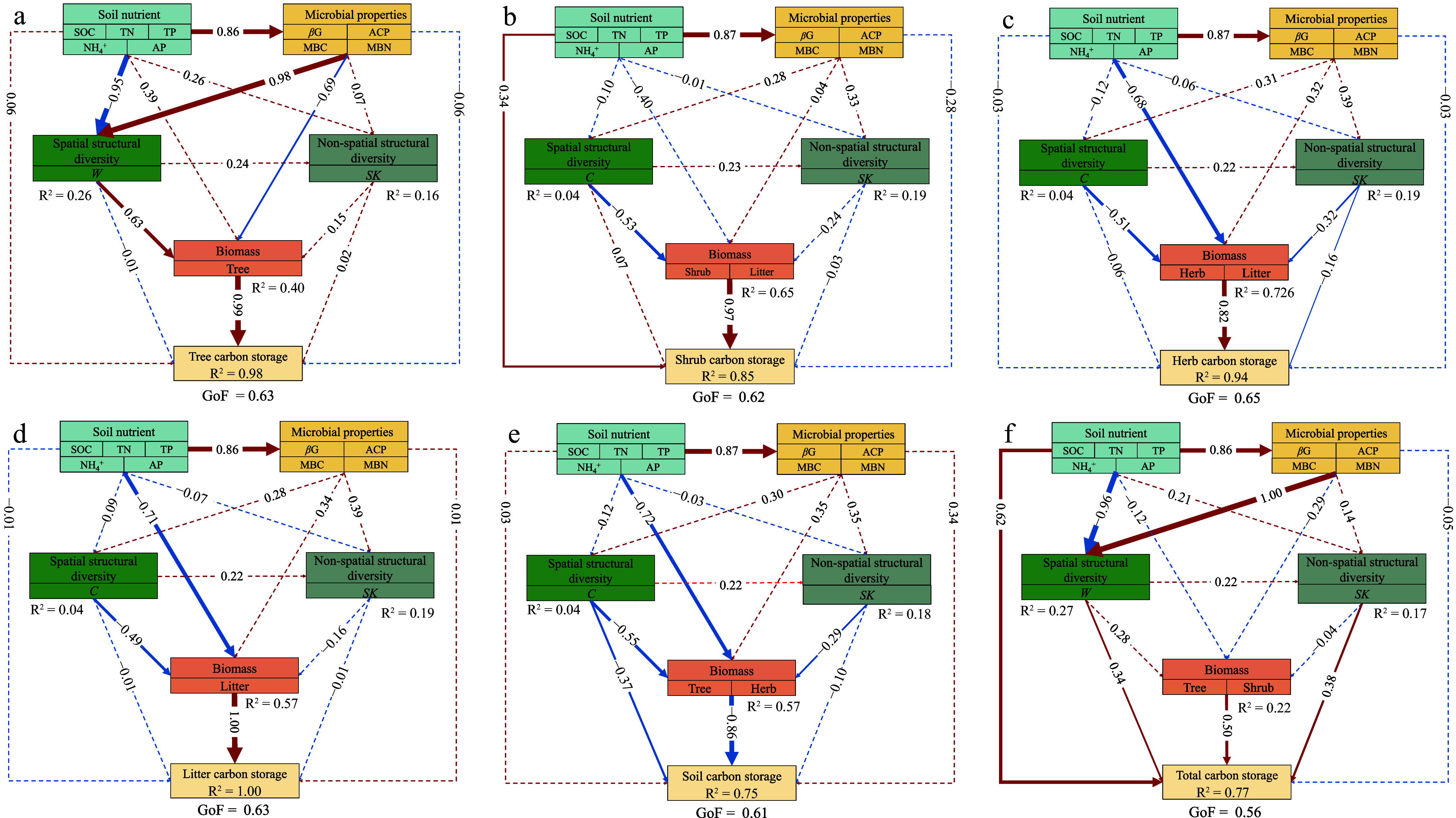
Predicted partial least squares path modeling (PLS-PM) for the direct and indirect impacts of the influencing factors on carbon storage. The results are shown for carbon storage in various components: (a) tree, (b) shrub, (c) herb, (d) litter, (e) soil, and (f) total carbon storage. In the structural model, the lines indicate paths, and the values adjacent to the lines denote the magnitude of the path coefficients calculated by PLS regression. R^2^ values are shown for all endogenous latent variables in the ellipses. The figure shows the final models after model diagnostic processes. The pseudo goodness-of-fit (GoF) of the models were shown, ranging from 0.56 to 0.65, implying that the predicted models fit well. The abbreviations of the influencing factors are explained in the Methods section.

The PLS-PMs were constructed to integrate the complex interrelationships among stand structural diversity, soil chemical and microbial properties, and biomass and carbon storage (Fig. 5). The stand spatial structural diversity indirectly enhances tree carbon storage through its positive influence on biomass (Fig. 5a). Conversely, the stand spatial structural diversity indirectly decreased carbon storage in the shrub, herb, and litter layers by negatively influencing biomass (Fig. 5b−d). The stand non-spatial structural diversity directly decreased carbon storage in the herb layer, as well as indirectly impacted it, by negatively influencing biomass (Fig. 5c). The stand spatial structural diversity directly increased soil carbon storage, and indirectly contributed to its increase by negatively influencing biomass. The stand non-spatial structural diversity indirectly decreased soil carbon storage by negatively influencing biomass (Fig. 5e). The stand spatial and non-spatial structural diversity showed direct effects on total carbon storage. Soil chemical properties and biomass had positive effects on total carbon storage (Fig. 5f).

## Discussion

### Differential roles of spatial and non-spatial structural diversity in shaping carbon pools

**Figure 6:**
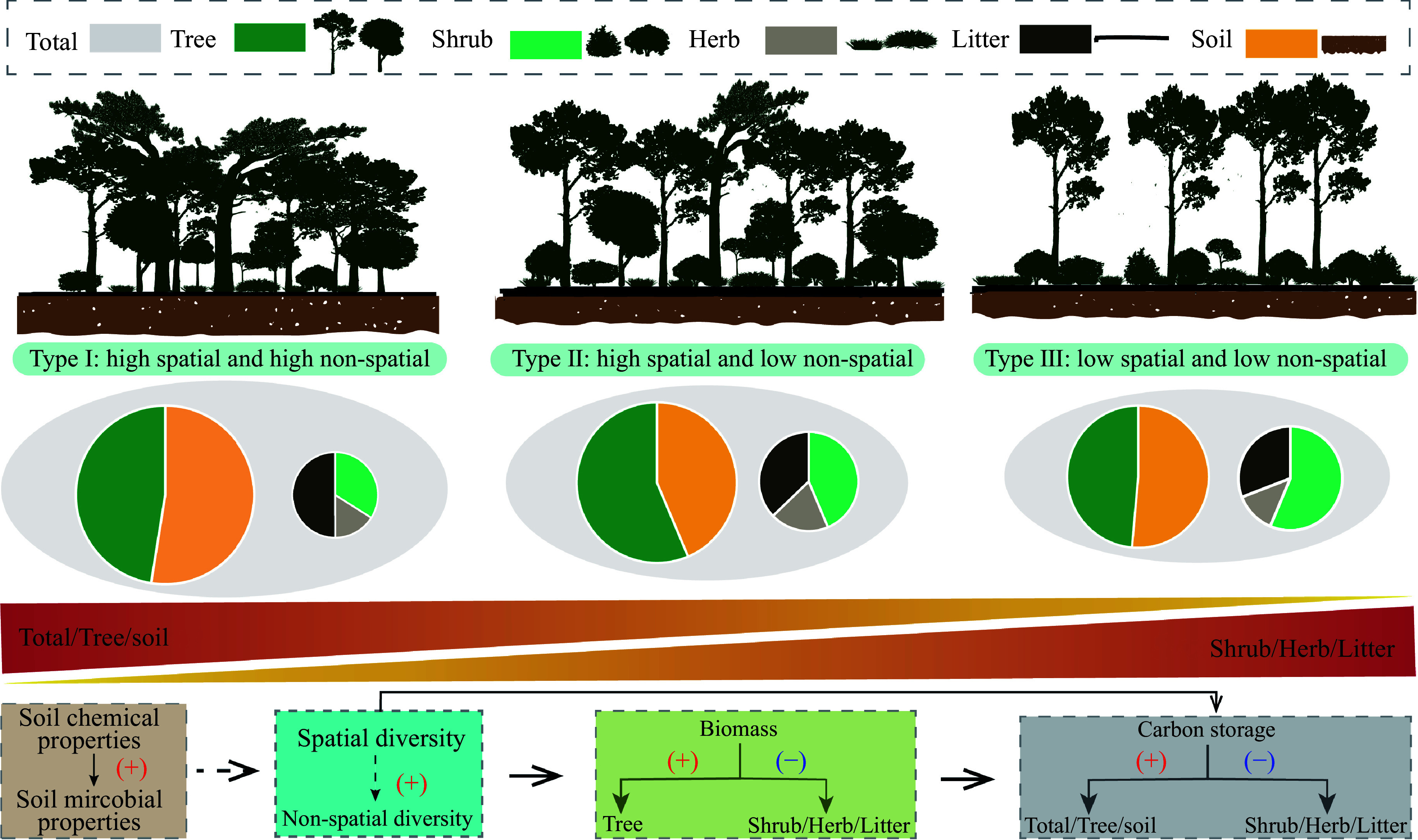
Concept diagram for the influences of structural diversity on carbon storage.

The PCA-classified stand types capture gradients of structural complexity. For instance, Type I, defined by high spatial and non-spatial structural diversity, features distinct vertical stratification and uneven horizontal arrangement. Such structural attributes reflect complex canopy organization and spatial heterogeneity, often linked to improved ecological performance^[2,3]^. Based on the PCA analysis results, our findings showed that stand structural diversity positively affects ecosystem carbon storage, such as overstory and soil layers, a conclusion that aligns with numerous previous studies focused on stand structural diversity^[9,30,31]^. This implies that an increase in stand structural diversity fosters the coexistence of trees with varied growth forms (e.g. broad-crowned vs. slender) and spatial distribution strategies (e.g. aggregated vs. dispersed) (Fig. 6). This coexistence facilitates the efficient utilization of spatial niches and light resources, aligning with the principles of niche complementarity theory^[2,32]^.

While past studies link stand structural diversity to carbon storage, this research pinpoints key stand spatial and non-spatial diversity indicators for different carbon pools^[1,3,33]^. Our findings further demonstrate that stand spatial structural diversity is more strongly associated with ecosystem carbon storage, including the carbon stored in tree, shrub, herb, and litter layers, compared to non-spatial structural diversity (Supplementary Fig. S2). According to niche complementarity theory, high stand spatial structural diversity, reflected by indicators such as the uniform angle index (capturing spatial tree arrangement), and dominance (representing size differentiation), fosters a complex vertical and horizontal distribution of trees^[2,34]^. This structural complexity minimizes canopy overlap and shading, optimizing light capture efficiency. Enhanced light utilization boosts photosynthetic activity, driving greater biomass accumulation and increased carbon storage within the ecosystem. By contrast, stand non-spatial structural diversity influences carbon sequestration through species intermixing and tree size variation, with the *SK* index serving as a key indicator of size distribution within stands^[7,30]^. In this study, the *SK* index values (0.47−1.97) indicate a dominance of large trees, which significantly shapes the distribution of light, water, and nutrients. This dominance suggests that a few large individuals contribute substantially to the community's biomass, play a pivotal role in determining the community's ecological function.

### Associations between stand structural diversity and carbon storage across multiple pools

In this study, indicators of stand structural diversity influencing the carbon storage of tree and soil layers differ significantly from those affecting the understory layer. As the dominant components of forest carbon storage, the tree and soil layers generally exhibit trends consistent with changes in total carbon storage. The tree layer, accounting for over 50% of total carbon storage in forest ecosystems^[35,36]^, plays a pivotal role in regulating ecosystem carbon dynamics. Among structural diversity indicators, the *W* index, which reflects spatial structural diversity, has been shown to enhance tree layer biomass and, consequently, its carbon storage. A high *W* index typically indicates that trees are distributed in small clusters with substantial spacing between them. This spatial arrangement minimizes direct competition and optimizes the use of light, water, and nutrients^[37−39]^. Additionally, the efficient resource allocation associated with a high *W* index reduces canopy overlap and shading, further improving light capture efficiency. These dynamics collectively enhance photosynthetic activity and biomass accumulation, contributing to greater carbon storage in the forest ecosystem.

Forest soil carbon reserves are highly sensitive to environmental changes, with stands exhibiting high spatial and non-spatial structural diversity demonstrating the highest carbon storage. Structural parameters, such as the *C* and *SK* indexes, indirectly influence soil carbon storage by regulating tree and herb biomass, with the herb layer playing a particularly significant role. The *C* index directly limits soil carbon storage by intensifying competition for resources like light and nutrients, which suppresses the growth of certain vegetation layers, particularly trees and herbs. However, this suppression redistributes resources to less competitive layers, such as the herb layer, thereby enhancing its contribution to soil organic matter inputs, including root exudates and decomposing plant material, which are vital for sustaining microbial activity^[40]^. The *SK* index, a non-spatial structural parameter, promotes heterogeneity within the stand, optimizing niche differentiation and resource use efficiency. This heterogeneity reduces interspecific and intraspecific competition, leading to a more balanced contribution of tree and herb biomass to soil carbon inputs. By supporting diverse vegetation dynamics, *SK* index indirectly facilitates a steady supply of organic matter to the soil, which enhances the microbial activity and nutrient cycling. These processes collectively improve the soil's capacity to retain carbon over extended periods^[41,42]^.

The overstory layer, as the dominant component of forest ecosystems, plays a crucial role in shaping the composition, species richness, and biomass of the understory, including shrubs and herbs. This influence is mediated by resource competition and structural effects driven by canopy architecture^[43,44]^. Our study reveals that increased structural diversity within the overstory is associated with a reduction in shrub and herb biomass, which in turn diminishes carbon storage in these layers and the litter layer. Dense tree distribution, reflected by a high *C* index, limits sunlight penetration to the understory, constraining photosynthesis and reducing biomass production in shrub and herb layers^[5]^. This reduction stems from the uneven light distribution caused by the high spatial structural diversity of the overstory, which limits solar radiation reaching understory vegetation^[45−47]^. Restricted light availability inhibits the growth of shade-intolerant shrub and herb species, leading to a decrease in biomass. As a result, the input of organic material, such as plant residues, into the litter layer is reduced, thereby weakening its capacity to temporarily store carbon due to a decline in decaying material accumulation^[48,49]^. Furthermore, this decrease in biomass and litter production disrupts nutrient cycling and organic matter recycling on the forest floor, amplifying the impact on ecosystem functioning.

Moreover, dense tree arrangements exacerbate root competition for water and nutrients, which not only suppresses the growth of understory vegetation, but also indirectly reduces carbon storage capacity. This finding aligns with previous studies showing that carbon storage in understory vegetation is negatively impacted by shading and competitive pressures from the overstory^[43,50,51]^. An increase in large trees benefits forest regeneration and genetic diversity by providing more mature, seed-producing individuals. However, overly dense large-tree populations may impede seed dispersal and suppress seedling establishment, potentially undermining long-term stand stability and biodiversity. These results highlight the delicate balance between overstory structural attributes and understory regeneration dynamics.

### Implications and limitations

Our findings emphasize the complex interplay between overstory structural diversity, and understory dynamics. Although enhanced overstory diversity supports species regeneration and strengthens ecological functioning, it can also reduce understory carbon storage by increasing resource competition and altering light availability. This dual effect highlights the importance of adopting adaptive management practices that balance carbon sequestration with ecosystem integrity. A combined approach involving selective thinning to reduce belowground competition, maintaining canopy complexity to support biodiversity, and creating small canopy openings of 20 to 40 square meters to temporarily improve understory light and resource conditions offers a practical solution. Such interventions require careful spatial planning to retain structural heterogeneity while enhancing understory function^[52−54]^. It is equally important to preserve legacy trees, which provide a continuous seed source essential for maintaining genetic diversity and ensuring long-term stand stability. Crucially, management regimes must preserve legacy trees to maintain a continuous seed supply, which is fundamental for sustaining the genetic diversity necessary for long-term stand stability. However, our findings suggest that when large-tree densities become excessive, they can inhibit seedling development, potentially offsetting the regenerative benefits these legacy trees provide.

The extent to which this study can be generalized is limited by two key considerations. First, the PCA-derived stand classification system, while methodologically transferable for structural diversity quantification, is ecologically specific to secondary subtropical Masson pine forests. The ecological interpretations, with a focus on stand structural diversity rather than species diversity metrics, are closely linked to the monospecific composition and mid-successional stage of these stands. Consequently, the framework may not capture carbon dynamics drivers in primary forests or mixed-species ecosystems, where species interactions and disturbance regimes differ fundamentally. Second, because the sample size is limited (n = 13 plots) and confined to a single bio-geographic region, uncertainties arise when attempting to extrapolate the findings to Masson pine forests across different climatic gradients, or to forests dominated by other tree species. This small-scale sampling may inadequately represent subtropical pine stand variability, while the single-region focus precludes assessment of broader biogeographic controls (e.g., edaphic or climatic factors) on structure-carbon relationships. Future studies should validate the framework through multi-regional plot networks and experimental manipulations (e.g., canopy gap gradients) across forest types with varying succession pathways.

## Conclusions

This study demonstrates that stand spatial structural diversity plays a more significant role than stand non-spatial structural diversity in regulating carbon storage in Masson pine forests. Stands with higher spatial structural diversity achieve greater total and overstory carbon storage, though this pattern does not extend to the understory layers, including shrub, herb, and litter. These findings indicate that increasing stand spatial structural diversity can enhance carbon sequestration, but may lead to a reduction in understory vegetation biomass. Considering the ecological importance of the understory layer, future research should focus on exploring strategies to optimize spatial structure for maximizing carbon storage, while supporting understory vegetation growth.

## SUPPLEMENTARY DATA

Supplementary data to this article can be found online.

## Data Availability

The datasets generated during and/or analyzed during the current study are available from the corresponding author on reasonable request.
