# Supplementary material for: Effects of stand structural diversity on carbon storage of Masson pine forests in Fengyang Mountain Nature Reserve, China
Source: For Res (Fayettev). 2025 Jun 6;5:e011. doi: 10.48130/forres-0025-0010 (PMC12441239; doi:10.48130/forres-0025-0010)
Supplement: Supplementary file 1 — Supplementary data to this article can be found online. [file FR-2025-5-0010-Supplementary.zip › 10.48130_forres-0025-0010-Suppl-TableS1.pdf]

Table S1 Basic information of sampling plots

| Plot | Stand type | Stand density (tree/ha) | Elevation (m) | Slope aspect | Slope degree (°) | Max DBH (cm) | Min DBH (cm) | Mean DBH (cm) | Tree Species richness | Tree Shannon-Weiner index | Tree Simpson's index | Species composition |
|---|---|---|---|---|---|---|---|---|---|---|---|---|
| 1 | II | 1475 | 355 | Southeast | 35 | 62 | 5.4 | 16.24 | 7 | 1.78 | 0.81 | ***Castanopsis sclerophylla*, *Symplocos stellaris*, *Pinus massoniana*, *Schima superba*, *Cyclobalanopsis glauca*, *Machilus nanmu*, *Castanopsis jucunda*,** *Ilex elmerrilliana*, *Loropetalum chinense*, *Eurya japonica*, *Litsea pungen*, *Lophatherum gracile*, *Dryopteris erythrosora*, *Dicranopteris pedata* |
| 2 | I | 1500 | 345 | East | 30 | 43.8 | 5 | 13.85 | 10 | 2.10 | 0.85 | ***Dalbergia hupeana*, *Castanopsis sclerophylla*, *Symplocos stellaris*, *Pinus massoniana*, *Castanopsis carlesii*, *Lithocarpus litseifolius*, *Cyclobalanopsis glauca*, *Machilus nanmu*, *Antidesma japonicum*, *Castanopsis jucunda*,** *Syzygium buxifolium*, *Symplocos lancifolia*, *Camellia fraterna*, *Itea chinensis*, *Eurya nitida*, *Ardisia japonica*, *Lophatherum gracile*, *Dryopteris erythrosora*, *Dicranopteris pedata*, *Alpinia japonica* |
| 3 | II | 1000 | 355 | Southeast | 35 | 45.7 | 5 | 18.22 | 7 | 1.83 | 0.82 | ***Ilex chinensi*, *Quercus aliena*, *Castanopsis sclerophylla*, *Pinus massoniana*, *Schima superba*, *Cyclobalanopsis glauca*, *Symplocos sumuntia*,** *Ilex elmerrilliana*, *Loropetalum chinense*, *Vaccinium duclouxii*, *Lophatherum gracile*, *Dryopteris erythrosora*, *Dicranopteris pedata*, *Woodwardia japonica*. |
| 4 | III | 700 | 349 | East | 38 | 57.2 | 6.5 | 19.29 | 6 | 1.76 | 0.82 | ***Castanopsis sclerophylla*, *Pinus massoniana*, *Cyclobalanopsis glauca*, *Machilus nanmu*, *Symplocos*** |

| | | | | | | | | | | | | |
|---|---|---|---|---|---|---|---|---|---|---|---|---|
| | | | | | | | | | | | | **sumuntia**, **Castanopsis jucunda**, *Diplospora dubia*, *Loropetalum chinense*, *Camellia cuspidata*, *Lasianthus japonicus*, *Eurya loquaiana* Dunn, *Lophatherum gracile* Brongn., *Alpinia japonica*, *Curculigo orchioides*, *Dicranopteris pedata*, *Woodwardia japonica*. |
| 5 | III | 875 | 340 | Southeast | 37 | 43.5 | 6.9 | 19.62 | 11 | 2.23 | 0.88 | **Eurya muricata**, **Photinia bodinieri**, **Castanopsis sclerophylla**, **Pinus massoniana**, **Lithocarpus litseifolius**, **Cyclobalanopsis glauca**, **Machilus nanmu**, **Elaeocarpus sylvestris**, **Symplocos sumuntia**, **Castanopsis jucunda**, **Elaeocarpus chinensis**, *Diplospora dubia*, *Symplocos lancifolia*, *Lindera chienii*, *Rhododendron latoucheae* Franch., *Myrsine seguinii*, *Lindera aggregata*, *Rhododendron simsii*, *Carex dimorpholepis*, *Dryopteris erythrosora*, *Dicranopteris pedata*, *Woodwardia japonica* |
| 6 | III | 1200 | 340 | Southeast | 33 | 43.8 | 5.1 | 14.07 | 10 | 1.94 | 0.81 | **Liquidambar formosana**, **Dalbergia hupeana**, **Liriodendron chinense**, **Pinus massonian**, **Schima superba**, **Cyclobalanopsis glauca**, **Machilus nanmu**, **Camellia japonica**, **Symplocos sumuntia**, **Castanopsis jucunda**, *Smilax china*, *Rhododendron simsii*, *Diplospora dubia*, *Lophatherum gracile*, *Dryopteris erythrosora*, *Dicranopteris pedata*, *Woodwardia japonica*, *Liriope spicata*. |
| 7 | II | 975 | 355 | East | 30 | 43.8 | 5.1 | 17.97 | 11 | 2.04 | 0.83 | **Diplospora dubia**, **Ilex elmerrilliana**, **Castanopsis sclerophylla**, **Pinus massoniana**, **Litsea pungens**, **Cyclobalanopsis glauca**, **Symplocos sumuntia**, **Aidia canthioides**, **Cyclobalanopsis myrsinifolia**, **Morella rubra**, |

| | | | | | | | | | | | | |
|---|---|---|---|---|---|---|---|---|---|---|---|---|
| | | | | | | | | | | | | ***Camphora officinarum*****,** *Yushania baishanzuensis*, *Syzygium buxifolium*, *Camellia cuspidata*, *Lindera aggregata*, *Eurya loquaiana*, *Eurya rubiginosa*, *Rhus chinensis*, *Camellia cuspidata*, *Chron.Loropetalum*, *Lophatherum gracile*, *Dryopteris erythrosora* Kuntze, *Dicranopteris pedata*, *Woodwardia japonica* |
| 8 | II | 1025 | 355 | East | 35 | 32.7 | 5.1 | 15.22 | 12 | 2.32 | 0.89 | ***Ilex chinensis*****,** ***Castanopsis tibetana*****,** ***Quercus aliena*****,** ***Lithocarpus glaber*****,** ***Castanopsis sclerophylla*****,** ***Pinus massoniana*****,** ***Castanopsis carlesii*****,** ***Schima superba*****,** ***Cyclobalanopsis glauca*****,** ***Machilus nanmu*****,** ***Camellia japonica*****.,** ***Camphora officinarum***, *Syzygium buxifolium*, *Symplocos lancifolia*, *Loropetalum chinense*, *Woodwardia japonica*, *Dicranopteris pedata* |
| 9 | I | 1650 | 365 | Northeast | 25 | 48.6 | 5.1 | 16.52 | 7 | 1.48 | 0.71 | ***Ilex chinensis*****,** ***Pinus massoniana*****.,** ***Castanopsis carlesii*****,** ***Schima superba*****,** ***Cyclobalanopsis glauca*****,** ***Camellia japonica*****,** ***Castanopsis eyrei*****,** *Symplocos anomala*, *Styrax odoratissimus*, *Diplospora dubia*, *Symplocos lancifolia*, *Vaccinium iteophyllum*, *Ilex pubescens*, *Lindera aggregata*, *Zanthoxylum armatum*, *Lophatherum gracile*, *Woodwardia japonica*, *Dryopteris erythrosora*, *Dicranopteris pedata*, *Alpinia japonica*. |
| 10 | I | 1550 | 360 | Southeast | 30 | 43.2 | 5 | 12.16 | 6 | 1.60 | 0.77 | ***Ilex chinensis*****,** ***Castanopsis sclerophylla*****,** ***Pinus massoniana*****,** ***Schima superba*****,** ***Cyclobalanopsis glauca*****,** ***Camellia japonica*****,** *Syzygium buxifolium*, *Loropetalum chinense*, *Ilex pubescens*, *Lindera aggregata*, *Lophatherum gracile*, *Dryopteris erythrosora*, *Dicranopteris pedata* |

| | | | | | | | | | | | | |
|---|---|---|---|---|---|---|---|---|---|---|---|---|
| 11 | I | 1050 | 365 | Southeast | 35 | 44.2 | 11.2 | 20.99 | 9 | 1.81 | 0.77 | ***Liquidambar formosana*, *Zelkova serrata*, *Castanopsis sclerophylla*, *Archidendron lucidum*, *Pinus massoniana*, *Lithocarpus litseifolius*, *Ligustrum lucidum*, *Cyclobalanopsis glauca*, *Camphora officinarum***, *Syzygium buxifolium*, *Loropetalum chinense*, *Ilex pubescens*, *Litsea pungens*, *Woodwardia japonica*, *Dryopteris erythrosora*, *Dicranopteris pedata*, *Phyllostachys aurea* |
| 12 | III | 1125 | 370 | Southeast | 32 | 39.7 | 5.1 | 15.52 | 8 | 1.95 | 0.85 | ***Diplospora dubia, Castanopsis sclerophylla*, *Pinus massoniana*, *Schima superba*, *Cyclobalanopsis glauca*, *Machilus nanmu*, *Castanopsis jucunda*, *Barringtonia racemosa***, *Syzygium buxifolium*, *Rhododendron simsii*, *Symplocos lancifolia*, *Camellia fraterna*, *Lindera glauca*, *Ardisia japonica*, *Lophatherum gracile*, *Woodwardia japonica*, *Vallisneria natans*, *Dryopteris erythrosora*, *Dicranopteris pedata*, *Alpinia japonica* |
| 13 | II | 1290 | 365 | Southeast | 28 | 41.1 | 5.2 | 18.17 | 4 | 1.13 | 0.64 | ***Liriodendron chinense*, *Pinus massoniana*, *Castanopsis carlesii*, *Cyclobalanopsis glauca***, *Loropetalum chinense*, *Ilex pubescens*, *Eurya hebeclados*, *Lindera aggregata*, *Eurya rubiginosa*, *Lophatherum gracile*, *Dryopteris erythrosora*, *Dicranopteris pedata* |

Note：Tree species is thickened in species composition.
