# Supplementary material for: Effects of stand structural diversity on carbon storage of Masson pine forests in Fengyang Mountain Nature Reserve, China
Source: For Res (Fayettev). 2025 Jun 6;5:e011. doi: 10.48130/forres-0025-0010 (PMC12441239; doi:10.48130/forres-0025-0010)
Supplement: Supplementary file 1 — Supplementary data to this article can be found online. [file FR-2025-5-0010-Supplementary.zip › 10.48130_forres-0025-0010-Suppl-TableS2.pdf]

Table S2 Calculation formula and description of stand structural diversity parameters

	Parameter	Abbreviation	Calculation formula	Description
Non-spatial structure diversity	Coefficient of variation	*CV*	$CV_{DBH} = \frac{SD_{DBH}}{\overline{DBH}}$	SD_{DBH} represents the standard deviation of DBH; $\overline{DBH}$ denotes the average diameter of the trees in the sample plot. A higher CV indicates a greater degree of tree size differentiation within the stand.
	Skewness of distribution	*SK*	$SK = \frac{\sum_{i=1}^{n}\left(DBH - \overline{DBH}\right)^3}{(n-1)SD_{DBH}^3}$	n represents the number of trees in the sample plot, and DBH stands for diameter at breast height. $\overline{DBH}$ is the average diameter of the trees in the sample plot. A higher absolute value of SK indicates a greater degree of size differentiation within the forest.
	Gini coefficient	*GC*	$GC = \frac{\sum_{i=1}^{n}(2i - n - 1)BA_i}{\sum_{i=1}^{n} BA_i(n - 1)}$	BA_i represents the basal area of a single tree. A GC value of 0 indicates an even distribution of tree sizes. The larger the GC, the more uneven the size or growth between trees. The higher the GC, the more uneven the size or growth between trees.
	Shannon-Weiner index	*H*	$H^{'} = -\sum (P_i ln P_i)$	The diameter intervals are set at 5 cm, with DBH ranging from 5 to 65 cm. Pi represents the total number of trees at each diameter level. $H^{'}$ is used to analyze tree size diversity.
Spatial structure diversity	Dominance	*U*	$U_i = \frac{1}{n}\sum_{j=1}^{n} K_{ij}$	If the jth neighboring tree is larger than the ith reference tree, K_{ij} = 1, otherwise K_{ij} = 0. DO describes the relative size of adjacent trees within a community structure unit. Four nearest neighboring trees and a reference tree were selected to form the spatial structure unit (as follows).
	Uniform angle index	*W*	$W_i = \frac{1}{n}\sum_{j=1}^{n} Z_{ij}$	The *W* represents the angle between neighbors. If $a < a_0$, Z_{ij} = 1; otherwise, Z_{ij} = 0. The W index indicates the spatial dispersion of the four nearest neighbors around the reference tree. Higher values indicate a transition from regular to random to clumped spatial patterns patterns.
	Stand Mingling	*M*	$M_i = \frac{1}{n}\sum_{j=1}^{n} V_{ij}$	If the jth neighboring tree is not of the same species as the ith reference tree, V_{ij} = 1, otherwise, V_{ij}= 0. A higher value indicates greater species diversity within the structural unit.
	Crowding index	*C*	$C_i = \frac{1}{n}\sum_{j=1}^{n} Y_{ij}$	If the jth neighboring tree's crown is connected to the ith reference tree's crown, Y_{ij}=1, otherwise, Y_{ij}=0. A higher value indicates greater tree density.
