# Supplementary material for: Effects of stand structural diversity on carbon storage of Masson pine forests in Fengyang Mountain Nature Reserve, China
Source: For Res (Fayettev). 2025 Jun 6;5:e011. doi: 10.48130/forres-0025-0010 (PMC12441239; doi:10.48130/forres-0025-0010)
Supplement: Supplementary file 1 — Supplementary data to this article can be found online. [file FR-2025-5-0010-Supplementary.zip › 10.48130_forres-0025-0010-Suppl-TableS3.pdf]

Table S3 Biomass regression model of tree species (groups)

Tree species and functional groups	Stem	Branch	Leaf	Root
Pinus massoniana	lnB=2.178×ln(DBH)-1.815	lnB=2.552×ln(DBH)-4.934	lnB=2.333×ln(DBH)-5.466	lnB=2.728×ln(DBH)-5.206
Cyclobalanopsis glauca	lnB=2.358×ln(DBH)-1.915	lnB=2.835×ln(DBH)-4.837	lnB=2.750×ln(DBH)-5.394	lnB=2.948×ln(DBH)-4.957
Schima superba	lnB=2.442×ln(DBH)-2.419	lnB=2.108×ln(DBH)-2.657	lnB=1.577×ln(DBH)-2.747	lnB=2.233×ln(DBH)-3.221
Deciduous broadleaved	lnB=2.501×ln(DBH)-2.772	lnB=2.691×ln(DBH)-4.724	lnB=2.599×ln(DBH)-6.234	lnB=2.282×ln(DBH)-3.272
Evergreen broadleaved	lnB=2.419×ln(DBH)-2.428	lnB=2.375×ln(DBH)-3.406	lnB=2.013×ln(DBH)-3.760	lnB=2.343×ln(DBH)-3.329

B represent biomass of tree species (groups).
