# Supplementary material for: Effects of stand structural diversity on carbon storage of Masson pine forests in Fengyang Mountain Nature Reserve, China
Source: For Res (Fayettev). 2025 Jun 6;5:e011. doi: 10.48130/forres-0025-0010 (PMC12441239; doi:10.48130/forres-0025-0010)
Supplement: Supplementary file 1 — Supplementary data to this article can be found online. [file FR-2025-5-0010-Supplementary.zip › 10.48130_forres-0025-0010-Suppl-TableS4.pdf]

Table S4 Carbon content of main tree species (groups)

Species(groups)	*Pinus massoniana*	Oaks	Hardwood	Softwood	Shrub	Herb	Litter
Carbon content	0.4596	0.5004	0.4814	0.4956	0.5000	0.3998	0.4967
