# Supplementary material for: Effects of stand structural diversity on carbon storage of Masson pine forests in Fengyang Mountain Nature Reserve, China
Source: For Res (Fayettev). 2025 Jun 6;5:e011. doi: 10.48130/forres-0025-0010 (PMC12441239; doi:10.48130/forres-0025-0010)
Supplement: Supplementary file 1 — Supplementary data to this article can be found online. [file FR-2025-5-0010-Supplementary.zip › 10.48130_forres-0025-0010-Suppl-TableS5.pdf]

Table S5 The soil chemical and microbial properties among stand types

Properties	0-20 cm layer			20-40 cm layer		
	I (n = 4)	II (n = 5)	III (n = 4)	I (n = 4)	II (n = 5)	III (n = 4)
pH	4.86 ± 0.07	4.81 ± 0.04	4.95 ± 0.06	4.94 ± 0.06	4.89 ± 0.02	4.99 ± 0.06
SOC	40.6 ± 7.11	27.4 ± 2.53	29.76 ± 4.63	31.07 ± 8.79	14.12 ± 2.4	17.79 ± 4.11
TN	2.63 ± 0.35a	1.76 ± 0.10b	1.87 ± 0.22ab	1.94 ± 0.42	1.00 ± 0.21	1.18 ± 0.25
TP	0.13 ± 0.02a	0.09 ± 0.01ab	0.09 ± 0.00b	0.12 ± 0.03	0.06 ± 0.01	0.06 ± 0.01
AP	2.05 ± 0.3	1.59 ± 0.11	2.45 ± 0.6	1.88 ± 0.33	1.33 ± 0.21	1.62 ± 0.44
NH_4^+	16.97 ± 2.04	11.55 ± 1.97	15.13 ± 1.22	14.26 ± 2.19a	6.88 ± 1.67b	8.88 ± 1.51ab
NO_3^-	2.59 ± 1.02	0.75 ± 0.39	0.83 ± 0.08	1.33 ± 0.54	0.59 ± 0.2	0.8 ± 0.12
NAG	8.21 ± 0.45	6.97 ± 0.86	6.76 ± 1.39	9.63 ± 0.37	8.7 ± 1.36	6.85 ± 1.98
LAP	9.73 ± 1.39	8.78 ± 1.01	10.2 ± 2.12	8.22 ± 1.44	8.19 ± 1.08	7.48 ± 1.88
BG	0.4 ± 0.06	0.31 ± 0.03	0.31 ± 0.02	0.33 ± 0.08	0.19 ± 0.02	0.2 ± 0.04
ACP	161.62 ± 15.75a	146.32 ± 9.5ab	116.5 ± 5.92b	127.4 ± 32.16	107.54 ± 2.32	82.1 ± 7.42
ALP	0.17 ± 0.02	0.16 ± 0.02	0.12 ± 0.01	0.16 ± 0.03	0.14 ± 0.01	0.1 ± 0.01
MBC	343.24 ± 44.06a	237.91 ± 14.06ab	197.78 ± 26.76b	256.5 ± 67.37	136.27 ± 27.06	174.84 ± 40.68
MBN	40.35 ± 6.94a	20.38 ± 2.42b	20.49 ± 2.59b	28.07 ± 2.91	14.85 ± 1.82	23.14 ± 8.08
MBP	2.88 ± 1.22	2.83 ± 0.51	6.36 ± 1.59	3.08 ± 0.25	3.76 ± 1.22	2.92 ± 1.37

SOC, soil organic carbon; TN, total nitrogen; TP, total phosphorus; AP, available phosphorus; NH_4^+, ammonium nitrogen; NO_3^-, nitrate nitrogen; BG, β-glucosidase; NAG, β-N-acetylglucosamine glycosidase; LAP, leucine aminopeptidase; ACP, acid phosphatase; ALP, alkaline phosphatase; MBP, microbial biomass phosphorus; MBC, microbial biomass carbon; MBN, microbial biomass nitrogen. The data are shown as the Mean ± SE. Different lowercase letters indicate significant differences at $p < 0.05$.
